# Priorities, needs and willingness of use of nerve stimulation devices for bladder and bowel function in people with spinal cord injury (SCI): an Australian survey

**DOI:** 10.1038/s41394-024-00628-3

**Published:** 2024-03-21

**Authors:** Vanesa Bochkezanian, Kelsey J. Henricksen, Benjamin J. Lineburg, Louis A. Myers-Macdonnell, Dennis Bourbeau, Kim D. Anderson

**Affiliations:** 1 School of Health, Medical and Applied Sciences College of Health Sciences Building 34 Office 1.02, Bruce Highway, CQUniversity Australia, Rockhampton North, QLD 4702 Australia; 2 The Joanna Briggs Institute, Faculty of Health and Medical Sciences, The University of Adelaide, Adelaide, SA 5000 Australia; 3 School of Health, Medical and Applied Sciences College of Health Sciences, CQUniversity Australia 1/1.04-2, University Dr, Bundaberg, QLD 4670 Australia; 4 Louis Stokes Cleveland Department of Veterans Affairs Medical Center, Cleveland, OH 44106 USA; 5 Cleveland FES Center, Cleveland, OH 44106 USA; 6 MetroHealth Rehabilitation Institute, MetroHealth System, Cleveland, OH 44106 USA; 7 Department of Physical Medicine and Rehabilitation, Case Western Reserve University School of Medicine, Cleveland, OH 44106 USA

**Keywords:** Urological manifestations, Rehabilitation

## Abstract

**Study design:**

Anonymous online survey

**Objectives:**

To investigate the priorities, needs and willingness to adopt nerve stimulation devices for managing neurogenic bladder and bowel function in people with spinal cord injury (SCI) living in Australia.

**Setting:**

Online survey of people living with SCI in Australia.

**Methods:**

This anonymous online survey used Qualtrics and was advertised via standard communication channels, such as advocacy groups representing the SCI community in Australia, social media, attending SCI sporting events and by word-of-mouth.

**Results:**

Responses from 62 individuals (32% female, 68% male) were included. Bladder emptying through urethra without catheter was the highest priority for bladder function. Reducing time required for bowel routines and constipation were the top priorities regarding bowel function. The highest concern for internal/implanted devices was the 4% chance of device surgical removal, while wearing wires under the clothes was the main concern for external devices. 53% of respondents were willing to trial an implanted nerve stimulation device, while 70% would trial an external device to improve and gain independence in bladder and bowel function.

**Conclusion:**

The findings of this study highlighted the potential role in which nerve stimulation can have in addressing bladder and bowel dysfunction in people with SCI, and have also identified that there was a need for Australian physiotherapists to evaluate their role in bladder and bowel dysfunction. Results from this study can help guide further research in nerve stimulation devices for bladder and bowel dysfunction in people with SCI.

**Sponsorship:**

n/a

## Introduction

Approximately 20,800 people are living with a spinal cord injury (SCI) in Australia [1, 2]. People living with SCI experience a number of health impairments, such as paralysis and secondary complications, such as spasticity, pressure sores and bladder and bowel dysfunction [3]. Bladder and bowel dysfunction affects 80% of people with SCI further resulting in medical complications, social isolation and adversely affecting quality of life [3], whilst depreciating the person with SCI’s dignity and privacy [4]. Some of the current methods to address this dysfunction, such as indwelling catheters, increases the risk of developing urinary tract infections (UTI), which can lead to re-hospitalisation and further health complications in people with SCI [4]. Despite the reduction in mortality secondary to urological dysfunction in previous decades, there is still a 13% mortality rate [5]. Regaining bladder and bowel function are consistently rated as top priorities in people with SCI and emerging person-centred and knowledge translational research approaches indicate neurogenic bladder and bowel dysfunction is still a substantially unmet clinical requirement in this population [6, 7].

Current bladder and bowel management in people with SCI includes a range of techniques [8, 9]. However, these methods usually result in many medical complications and also require adequate hand dexterity or the help of a caregiver, further reducing their independence and impacting their physical and mental health [10, 11]. Therefore, new methods to reduce bladder and bowel dysfunction in people with SCI are extremely needed. One solution to address neurogenic bladder and bowel dysfunction in people with SCI is the use of nerve stimulation devices, which aim to stimulate or modulate the reflexes that control bladder, bowels, and musculature of the pelvic floor [12]. External nerve stimulation is a non-invasive novel form of peripheral nerve stimulation that involves an external device attached to the skin [12]. Internal nerve stimulation consists of a small device which is surgically implanted in the body to stimulate targeted nerves with mild electrical impulses [13]. The use of Transcutaneous Electrical Stimulation (TES) has been demonstrated to be effective at improving bladder and bowel dysfunction in people with SCI [12]. However, most of these studies have not used a person-centred approach and have not asked people with SCI about their willingness to use these types of nerve stimulation.

An understanding of the needs and priorities for bladder and bowel dysfunction and willingness to use nerve stimulation devices to address these dysfunctions in people with SCI have already been investigated within North America [6]. However, this evidence has not been gathered in people living with SCI in Australia. This evidence is important, as healthcare access, standards of care, access to technology and cost barriers may differ based on where individuals live (e.g. USA or Australia) and identifying some of these differences can inform potential solutions for individuals with SCI living in different parts of the world. For example, the proportion of health insurance in the USA is lower than other highest-income countries (i.e. Australia) [14], and this may need to be considered in the management of bladder and bowel in people with SCI living in different geographical locations. Considering that the need for a person-centred approach in SCI research has strongly been recommended to improve the chances of finding the right solutions to their main concerns [15], it is crucial to gather a person-centred understanding of Australians living with SCI. This will inform local stakeholders, clinicians and government organisations, so they can develop relevant solutions for bladder and bowel dysfunction in people with SCI living in Australia.

Therefore, the main aim of this study is to identify the priorities and needs voiced by people with SCI living with bladder and bowel dysfunction in Australia. The second aim of this study is to identify their potential willingness to adopt a nerve stimulation intervention for management of their bladder and bowel function.

## Methods

### Trial design

Anonymous online survey.

### Setting

Online survey of people living with spinal cord injury in Australia.

### Ethical approval

This study was approved by the Human Research Ethics Committee Research Division of Central Queensland University, reference 0000022316. We certify that all applicable institutional and governmental regulations concerning the ethical use of human volunteers were followed during this research.

### Participants

Inclusion criteria required for participants to be over 18 years of age and living in Australia with spinal cord injury (SCI).The target audience was reached by contacting spinal cord associations and other relevant Australian entities. Participants completed this online survey between 22nd June, 2020 and 21st August 2022.

### Survey design

A previously developed survey created by the North American Spinal Cord Consortium consumer advisory board was adapted to Australian-based individuals with SCI, obtaining authorisation from the original authors [6]. This survey was disseminated through Qualtrics (Qualtrics XM, Provo, USA, 2022, available at https://www.qualtrics.com) and was advertised via standard communication channels, such as advocacy groups representing people living with SCI in the community, social media and by word of mouth all across Australia and attending wheelchair sporting events in Queensland.

Snowball sampling was used to engage hard-to-reach communities, such as people with SCI [16].

The full survey is included as part of the supplement to this manuscript.

Survey data processing and statistical analysis were performed on SPSS Statistics software (Version 22.0, IBM, New York). A summary of statistics was provided numerically and graphically.

## Results

### Demographics

This study collected 62 responses from participants who indicated they were living with SCI in Australia and were over 18 years of age. All clinical data was self-referred. Table 1 summarises the demographic data of the survey respondents.

**Table 1:** Respondent demographics.

Gender	Male	68%	*N* = 42
	Female	32%	*N* = 20
Age	18–30	11%	*N* = 7
	31–45	35%	N = 22
	46–60	35%	*N* = 22
	61-75	16%	*N* = 10
	76+	2%	*N* = 1
Geographical location	Metropolitan	56%	*N* = 35
	Non- Metropolitan	44%	*N* = 27
Injury type	Traumatic	79%	*N* = 49
	Non-traumatic	19%	*N* = 12
	Not Sure	2%	*N* = 1
Years since SCI	Less than 1 year	6%	*N* = 4
	1–5 years	34%	*N* = 21
	6–10 years	10%	*N* = 6
	11–15 years	13%	*N* = 8
	16–20 years	8%	*N* = 5
	Over 20 years	29%	*N* = 18
Level of injury	Cervical - C1-4	20%	*N* = 13
	Cervical - C5-8	28%	*N* = 18
	Thoracic OR Lumbar OR Sacral - T1-S5	52%	*N* = 34
Severity of impairment	Complete	34%	*N* = 22
	Some Sensation	32%	*N* = 21
	<50% antigravity strength	15%	*N* = 10
	>50% antigravity strength	15%	*N* = 10
	Unsure	3%	*N* = 2
Performing transfers	Full Assistance	56%	*N* = 35
	Some Assistance	26%	*N* = 16
	No Assistance	18%	*N* = 11
Hand function	Complete Hand Function	63%	*N* = 39
	Some Hand Function	31%	*N* = 19
	No Hand Function	6%	*N* = 4
Leaving the house	Daily	54%	*N* = 33
	Weekly	31%	*N* = 19
	Fortnightly	5%	*N* = 3
	Monthly	7%	*N* = 4
	Never	3%	*N* = 2
Transportation barriers	Yes	11%	*N* = 7
	No	89%	*N* = 55
Financial barriers	Yes	16%	*N* = 10
	No	76%	*N* = 47
	Would rather not say	8%	*N* = 55
Sources of healthcare information			
	Health care providers	84%	*N* = 52
	Educational organisation	10%	*N* = 6
	Support groups	26%	*N* = 16
	Internet	34%	*N* = 21
	Friends	15%	*N* = 9
	Information sessions	10%	*N* = 6
	Other	3%	*N* = 2
Healthcare professionals Consulted			
	Physiatrist	2%	*N* = 1
	Neurologist	11%	*N* = 7
	Neurosurgeon	5%	*N* = 3
	Urologist	45%	*N* = 28
	Gastroenterologist	3%	*N* = 2
	Primary Care Physician (GP)	44%	*N* = 27
	Nurse	45%	*N* = 28
	No access to a health professional	6%	*N* = 4
	Other	15%	*N* = 9

The majority of respondents were male (68%). A majority of respondents lived in metropolitan areas (56%). Most (86%) respondents were aged 31–75 years, whilst 11% were aged 18–30 years.

This survey results indicated that 77% of respondents commonly consulted with general practitioners, urologists, and nurses to assist with bladder and bowel management. Additionally, generalised information regarding bladder and bowel management was received through their healthcare providers and the internet, with other important resources consisting of social support and advocacy organisations. In comparison, it was evident that within our study that there was minimal access to bowel specialists, with only 2% of respondents being reviewed by gastroenterologists.

The main severity of impairment reported by respondents was a complete lack of motor and sensory function below the level of injury, including the anal area (which correlates to what is defined as a complete SCI) (refer toTable 1).

### Bladder function

**Fig. 1:**
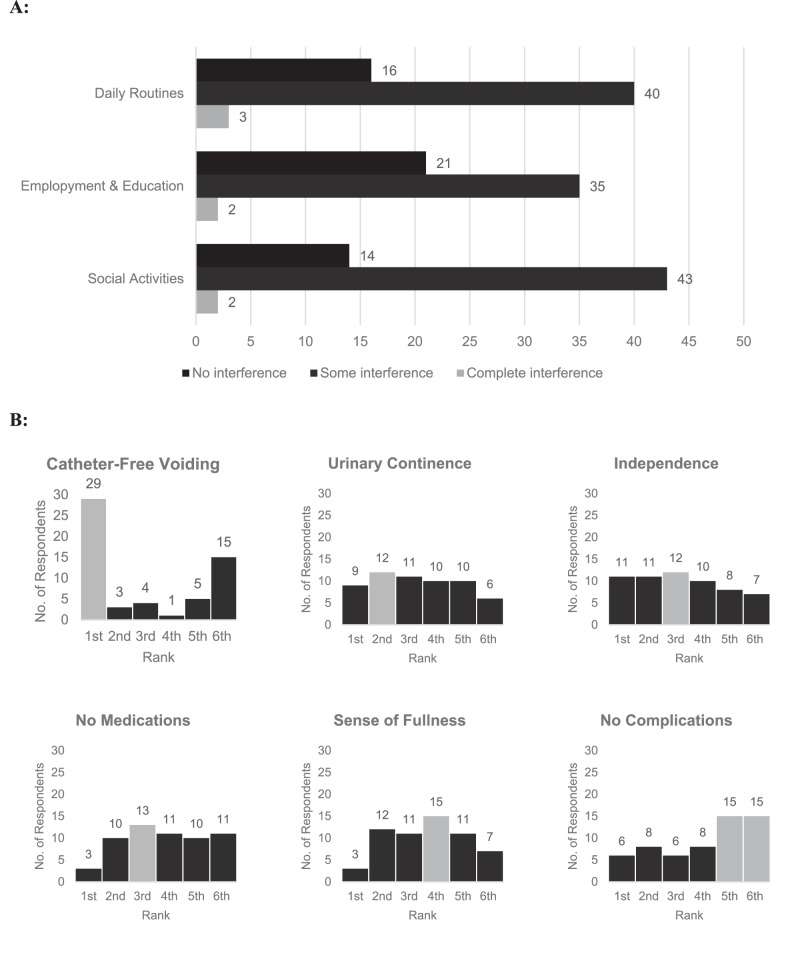
Bladder management. **A** Interference from Bladder Management: Respondents rated interference from bladder management on everyday activities as either, “no interference”, “some interference” or “complete interference”. **B** Ranked Priorities for Bladder Function Restoration: Respondents ranked from 1st to 6th, their priorities for the benefits of restoring bladder function. For each bladder function priority, the distribution of the rank scores that it received are shown.

Most respondents (93%) required some form of bladder management and 13% used more than one method for such purpose (refer to Table 2). Intermittent catheterisation via urethra was the most common method (47%) used to manage bladder function. Daily medication was the most common treatment used in combination with other methods (63%). Some or complete disruption to daily routines due to their bladder was reported by 73% repondents, whilst interruption to participation in education and/or employment was reported by 64%, and activity interference with family, neighbours and social groups by 76% of respondents. Just under half of respondents emptied their bladder between five and six times per day, with approximately one quarter of respondents emptying their bladder more than six times per day. Urinary tract infections (UTI) were the most common complication (67%) reported in the past 12 months, followed by urinary incontinence and autonomic dysreflexia (AD). Of the respondents reporting complications, 62% have experienced two or more complications and 36% have experienced three or more complications (Table 2). Emptying their bladder through the urethra without catheters was the highest reported priority, followed by independently managing their bladder and improving urinary incontinence (Fig. 1).

**Table 2:** Bladder management.

Bladder emptying frequency	Not applicable	10%	*N* = 6
	1–2 times/day	3%	*N* = 2
	3–4 times/day	17%	*N* = 10
	5–6 times/day	46%	*N* = 27
	6 + times/day	24%	*N* = 14
Daily bladder medications	Yes	63%	*N* = 38
	No	37%	*N* = 22
Assistance with bladder	Full assistance	10%	*N* = 6
	Some assistance	5%	*N* = 3
	No assistance	85%	*N* = 51
Assistance if incontinent	I do not experience urinary incontinence	18%	*N* = 11
	No assistance	42%	*N* = 25
	Some assistance	22%	*N* = 13
	Full assistance	18%	*N* = 11
Sense when bladder is full	Yes	73%	*N* = 44
	No	27%	*N* = 16
If bladder sense, avoid incontinence	Not Applicable - No sense of Fullness	28%	*N* = 17
	Yes	48%	*N* = 29
	No	23%	*N* = 14
Bladder management methods			
	I do not use any equipment	8%	*N* = 5
	Indwelling (Foley) catheter via urethra	7%	*N* = 4
	Condom catheter	7%	*N* = 4
	Suprapubic catheter	25%	*N* = 15
	Electrical stimulation	0%	*N* = 0
	Intermittent catheterisation via urethra	53%	*N* = 32
	Intermittent catheterisation via abdomen	0%	*N* = 0
	Bag on abdomen	2%	*N* = 1
	Absorbent pads or diapers	10%	*N* = 6
	Other	2%	*N* = 1
Complications associated with bladder management in last 12 months			
	I do not experience complications	18%	*N* = 11
	Clogged catheter	25%	*N* = 15
	Urinary tract infection	67%	*N* = 40
	Urinary incontinence	43%	*N* = 26
	Bladder or kidney stones	13%	*N* = 8
	Kidney disease, kidney failure or both	0%	*N* = 0
	Autonomic Dysreflexia (AD)	32%	*N* = 19
	Other	5%	N = 3

### Bowel function

This study’s results showed that 74% of respondents required at least 30 min or more to effectively complete their bowel routine. 70% of all respondents used multilple forms of bowel management strategies. Assistance with bowel management, digital stimulation, enemas, and laxatives accounted for 62% of reported management strategies, with 57% using medication (Table 3). Of those reporting to experience faecal incontinence (68%), only 32% required either some or full assistance. Just over half (53%) of respondents stated that their body gives them a conscious indication of bowel fullness indicating that a bowel movement was required, however only half (25%) of these respondents reported sufficient time to avoid an episode of incontinence.

**Table 3:** Bowel management.

Bowel routine duration	Not applicable	2%	*N* = 1
	Less than 30 min	25%	*N* = 15
	30–60 min	53%	*N* = 31
	1–2 h	14%	*N* = 8
	Greater than 2 h	7%	*N* = 4
Daily bowel medication	Yes	57%	*N* = 34
	No	43%	*N* = 26
Assistance with bowel	Full assistance	20%	*N* = 12
	Some assistance	12%	*N* = 7
	No assistance	68%	*N* = 41
Assistance if incontinent of stool	I do not experience faecal incontinence	8%	*N* = 5
	No Assistance	28%	*N* = 17
	Some Assistance	32%	*N* = 19
	Full Assistance	32%	*N* = 19
Sense when bowel is full	Yes	53%	*N* = 32
	No	45%	*N* = 27
	Not applicable—I use a colostomy bag	2%	*N* = 1
If bowel sense, avoid incontinence	N/A—no sense of fullness	35%	*N* = 21
	Yes	33%	*N* = 20
	No	32%	*N* = 19
Bowel management methods			
	Special diet	18%	*N* = 11
	Laxatives	43%	*N* = 26
	Enemas	47%	*N* = 28
	Suppositories	22%	*N* = 13
	Digital stimulation	60%	*N* = 36
	Manual evacuation (including transanal irrigation)	33%	*N* = 20
	External adaptive devices	3%	*N* = 2
	Implanted devices	0%	*N* = 0
	Colostomy bag	3%	*N* = 2
	Other	12%	*N* = 7
Complications associated with bowel management in last 12 months			
	I do not experience complications with my bowel	2%	*N* = 1
	Faecal incontinence	68%	*N* = 41
	Constipation	62%	*N* = 37
	Autonomic dysreflexia (AD)	23%	*N* = 14
	Haemorrhoids	57%	*N* = 34
	Bleeding	47%	*N* = 28
	Loose stool	64%	*N* = 38
	Complications with colostomy stoma	2%	*N* = 1
	Other	0%	*N* = 0

**Fig. 2:**
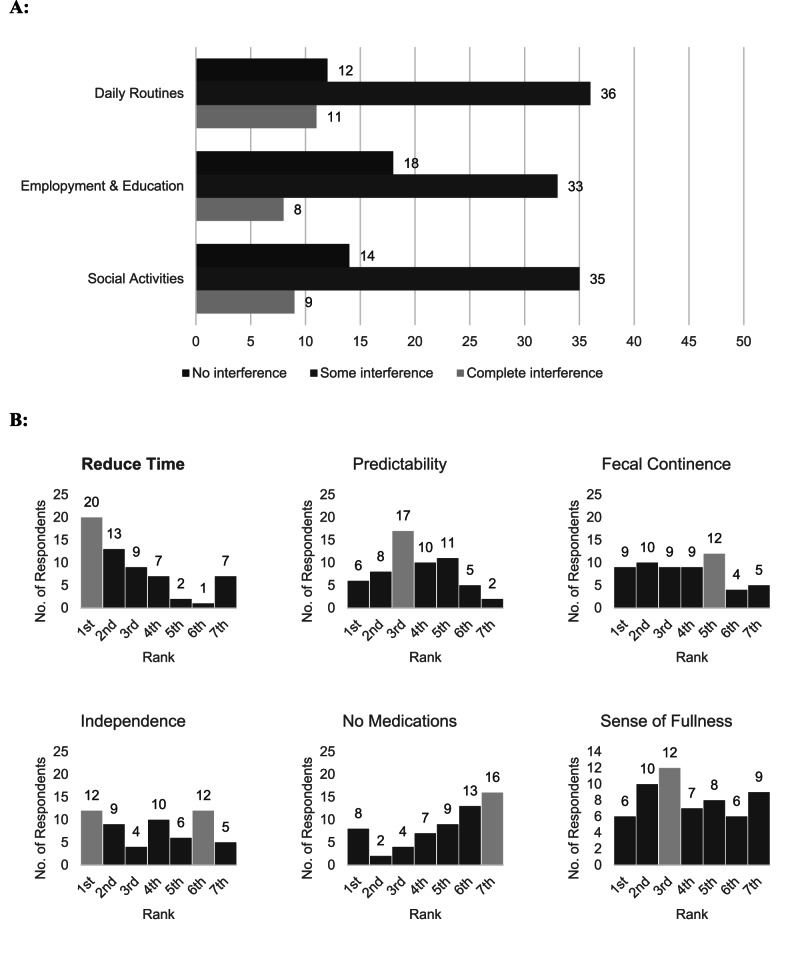
Bowel management. **A** Interference from Bowel Management: Respondents rated interference from bowel management on everyday activities as either, “no interference”, “some interference” or “complete interference”. **B** Ranked Priorities for Bowel Function Restoration: Respondents ranked from 1st to 6th, their priorities for the benefits of restoring bowel function. For each bowel function priority, the distribution of the rank scores that it received are shown.

Almost all (99%) of respondents reported experiencing complications with their bowel in the last 12 months because of their current management. Faecal incontinence was the most common complication followed by loose stools and constipation (Table 3). Figure 2A shows that 80% of respondents stated that they experienced some or complete disruption to their daily routine because of their bowels. Over a third of respondents (34%) rated reducing the time required for their bowel routine and reducing constipation as their top priority, followed by independence in bowel management (20%) (Fig. 2B).

### Willingness of use of nerve stimulation devices for bladder and bowel management

Over half of the respondents (53%) stated that they would be willing to trial internal stimulation devices when knowing the risks and benefits, and if it meant a reduction in medical complications related to bladder and bowel function. Similarly, when questioned about the likelihood of utilising an external nerve stimulation device, over 70% stated that they would be willing to trial the device if it resulted in improvement of overall independence of bladder and/or bowel management.

**Fig. 3:**
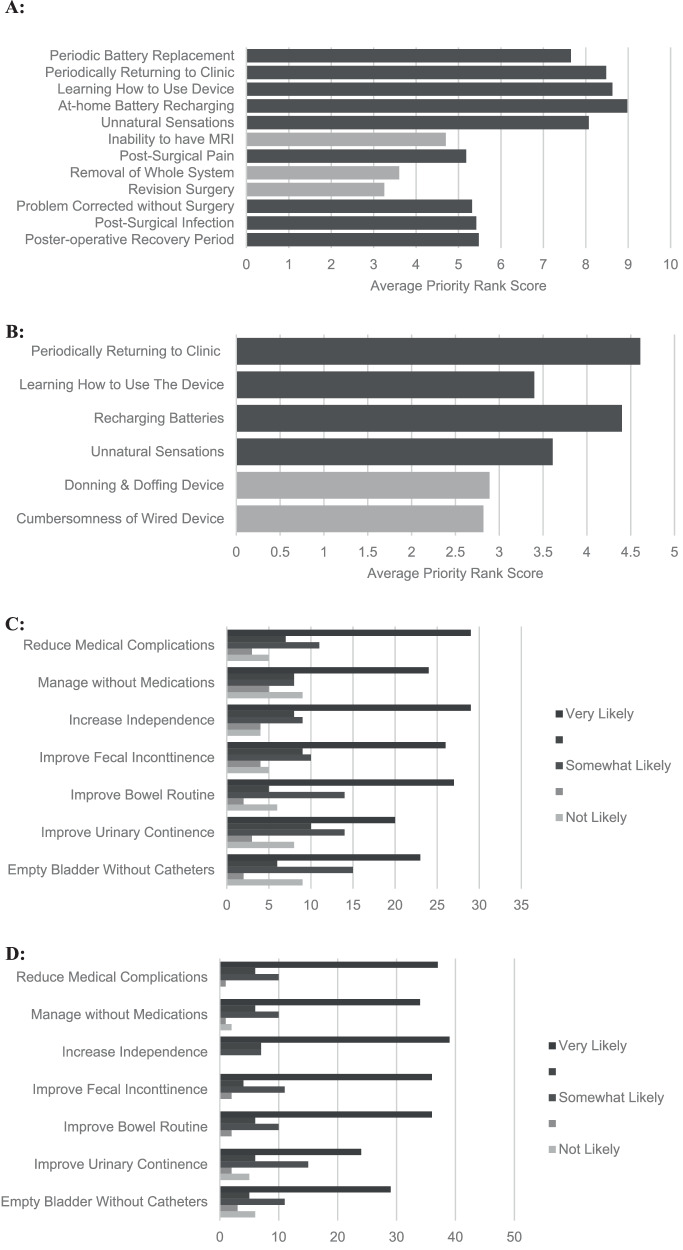
Concerns, risks and benefits associated with use of nerve stimulation devices for bladder and bowel management. **A** Ranked Concerns Associated with Implanted Device. **B** Ranked Concerns Associated with External Device: Respondents ranked their concerns associated with implanted device (*A*) and external device (*B*). Concerns were ranked from 1 to 6 for internal device and 1 -12 for implanted device with 1 representing most concerning and 6 (*A*) or 12 (*B*) representing least concern. The average rank for each concern is shown in Fig. 3A, B. **C** Likelihood of Accepting Implanted Device. **D** Likelihood of Accepting External Device. For each of the 7 potential benefits, respondents rated their likelihood of accepting an implanted device (*C*) after knowing the risks and benefits or an external device (*D*). The likelihood based on each benefit was rated from 1 to 5, where 1 represents “not likely”, and 5 represents “very likely”.

When respondents were asked about the use of an external nerve stimulation device, the two greatest concerns were (1) wearing a device with wires connecting to electrodes on the skin under the clothes and (2) the inconvenience of having to learn how to use the device with their current management strategies (Fig. 3B). The highest concern (32%) regarding the use of an internal stimulation device, was the 4% chance of requiring surgical removal of the whole system and the second greatest concern was not being able to have Magnetic Resonance Imaging (MRI) for life (Fig. 3A).

## Discussion

This study summarised the information gathered in a person-centred online survey related to the management and challenges of bladder and bowel functions in people with SCI in Australia. Nurses and urologists were the most consulted health care professionals to help with management of bladder and bowel function in people with SCI. However, when compared to a similar survey conducted in North America, respondents were referred to urologists (70%), primary care physicians (60%) and nurses (22%) [6], while this study’s respondents showed an even distribution of these three professionals (urologists 45%, GPs 44% and nurses 45%). There were other similarities between this previous study, as the main sources of information for bladder and bowel management were received by health care providers and via the internet. However, the main difference in our study’s results was that repondents were referred primarily to nurses for their bladder and bowel management. This highlights the difference in the healthcare systems in the USA and Australia, with the primary care physician being the entry point of all care in the USA [14], while Australians receive treatment from professional nurses, specially in rural and remote areas to mitigate long waiting times to consult medical doctors [17]. This result implied that healthcare professionals, especially nurses and online social support groups would be the main providers of information to people with SCI living in Australia, especially related to new information on nerve stimulation devices for bladder and bowel management.

Results from this study and a previous study completed in North America indicated that respondents did or could not frequently consult bowel care specialists, especially gastroenterologists for their bowel dysfunction [4]. Within Australia, gastrointestinal problems are responsible for a disproportionate amount of morbidity in people with SCI [18]. Results from this study indicated that having access to a gastroenterologist and educating about the importance of accessing these services during acute and chronic stages would allow implementation of tailored evidence-based treatments and effectively reduce the high morbidity rate related to these gastrointestinal problems in people with SCI [18, 19].

Considering that bowel management is part of the basic training for Physical Medicine and Rehabilitation Physicians (PM&R or physiatrist), our data of only one person consulting to a physiatrist also suggested an inadequate number of specialised staff, such as PM&R available to be consulted by people with SCI living in Australia. Our study showed physiotherapists were not a primary source of information for people with SCI living in Australia for bladder and bowel management. However, a study performed in Germany showed different results, showing that general practitioners and physiotherapists were the first point of contact after discharge, for providing useful resources for management of secondary complications, such as bladder and bowel dysfunction [20]. The difference between this current study’s results and results from the German study may suggest that Australian’s living with SCI in regional and rural areas may not get information for their bladder and bowel management due to difficulties accessing not only PM&R physicians, but also allied health professionals, such as specialised physiotherapists [21].

Most people with SCI living in Australia have access to healthcare and regular and free consultations with their General Practitioners (GPs) through government funding (i.e. NDIS) [22], however this access is different for people living in regional, remote and rural areas of Australia [23], and as such, outreach SCI-specialised clinics are often available in most states, such as Queensland, New South Wales, Victoria and South Australia. However, these outreach clinics have certain selection criteria, which excludes most people living outside of 200 km around the main metropolitan hospitals. Thus, one solution could be that Australian physiotherapists and other allied health professionals, who live and work in regional areas, should re-consider their role and seek further specialised training from nurses to be capable of providing up-to-date information on management of bladder and bowel in people with SCI. Another solution could also be that more SCI-speciliased allied health professionals were to be deployed to regional, remote and rural areas of Australia, considering its widespread geography and difficulties in healthcare access in those areas [23].

### Bladder function

The majority of this study’s respondents reported at least one complication resulting from their current bladder management in the past 12 months. Complications included UTI, urinary incontinence, autonomic dysreflexia (AD), clogged catheters and/or bladder or kidney stones. Comparatively, a previous study [24] indicated that urinary incontinence and UTI were upon the most commonly report comorbidities in people with SCI. Another previous study reported that UTI and urological reasons because of complications of catheterisation were two of the top five most common causes of rehospitalisation in people with SCI [18]. This indicates that if an alternate treatment method such as nerve stimulation were to be used, the prevalence of urinary complications due to catheterisation may be reduced, therefore, resulting in fewer re-hospitalisations.

This study’s results indicated that having control over bladder and avoiding episodes of urinary incontinence may reduce the frequency of bladder emptying. Thus, reducing hyperactivity of the bladder using electrical stimulation of sacral afferent nerves (an internal nerve stimulation device) may help reduce these episodes of urinary incontinence. This could contribute to an improved social engagement in people with SCI [25], seen to be affecting a large percentage of this study’s respondents. If nerve stimulation were to be used at home, the rate of leakage may decrease between catheterisations and the catheterised volume may increase significantly, which would reduce the need for assistance and the risk of UTI.

Considering that emptying the bladder without a catheter, independence with bladder management and improving the level of incontinence were ranked the three highest priorities by respondents in this study, a solution to these problems seems imperative. This was demonstrated in a previous study [24] where dorsal penile stimulation (a type of external stimulation) was successfully trialled in people with SCI. Thus, if nerve stimulation were to be used, fewer episodes of incontinence may have been experienced, reducing the level of assistance required and resulting in increased independence in people with SCI experiencing bladder dysfunction. However, based on this study’s survey, improving the donning and doffing and reducing the cumbersomeness of the wired devices would be essential components to be addressed in future design of external stimulation devices.

### Bowel function

Results about disruption to daily routine and participation in education/employment and interference with social activities as a result of bowel dysfunction were similar to a previous study [19], which showed a reduction in social engagement in people with SCI, which reduced access to social support and relationship development, potentially compromising physical and mental health and well-being.

Most of this study’s respondents required at least 30 min to complete their bowel routine, thus less time spent completing bowel routines would allow for more time socialising within the community as well as more willingness to access the community, promoting an increase in social engagement and relationship development, which is crucial to the well-being and quality of life of people with SCI [25]. A previous study showed evidence that external nerve stimulation can improve bowel management for individuals with tetraplegia and reduce the overall time spent completing a bowel routine [26]. Thus, if nerve stimulation was to be utilised, more time could be spent in participating in social community activities.

Based on this study’s results, the adverse impacts neurogenic bowel dysfunction has on people with SCI was evident, with numerous challenges and dependency on family members or carers for assistance to manage. This was consistent with Bourbeau et al.’s study [6], which reported that the lack of available treatment options for improving bowel function and bowel care duration after SCI interferes with their quality of life and independence.

This study’s results suggested faecal incontinence to be the most common complication, closely followed by loose stool and constipation. Additionally, this study’s results showed that reducing the time required for their bowel routine may also be impacting their ability to participate in social events within the wider community. Thus, these results showed that an alternative method for managing faecal incontinence would improve quality of life and participation in social activities in people with SCI suffering from bowel dysfunction [25]. An alternative method to address bowel incontinence can be a wearable implantable stimulation device, such as sacral nerve stimulation implantable device. This device has been reported a marked improvement in complete recovery of bowel incontinence 35-months following the implantation of sacral nerve stimulation and showed a significant improvement in quality of life on all scales among respondents who received the permanent implant 12 and 24 months after the surgery [27].

This study’s respondents reported the use of medication, but no use of nerve stimulation devices for bladder and bowel management. These results were different from Bourbeau et al.’s study [6], which identified participants used external and implanted devices along with colostomy bags for bowel management. However, in that previous study these were reported to be the least common used method. These combined responses can be attributed to the lack of information and promotion of nerve stimulation devices for bladder and bowel management, which may be the very promising results of these devices shown in research, but its clinical application stage being in its infancy stage [28].

### Willingness of use of nerve stimulation devices for bladder and bowel management

Given the potential benefits, nerve stimulation devices can improve bladder and bowel outcomes and respondents were willing to adopt both internal and external nerve stimulation devices. Among the concerns in adopting the internal devices, surgical removal of the implanted nerve stimulation device was the main one. However, due to the ability to achieve greater independence, there was a significant percentage of respondents willing to use the internal device. These findings were similar when comparing the responses from another similar previous study, in which respondents were willing to accept nerve stimulation devices to improve independence in management of their bladder and bowel [6]. One of the main differences with this study’s results were that Australians who indicated willingness to use an internal device would prefer avoiding repeated surgical interventions and would prefer to retain the ability to have an Magnetic Resonance Imagining (MRI). This suggests that the potential health consequences of repeated surgeries and not being able to have an MRI if needed are considered significant negative factors for people with SCI living in Australia.

However, in Bourbeau et al.’s study there was a more favourable trend towards external nerve stimulation devices, especially if they were designed with minimal visibility and not many electrodes attached to their skin [6, 29]. Therefore, further research should investigate the design of external devices, focusing on minimal visibility and improving usability (i.e easier donning and doffing and wireless devices).

Additionally, based on this study and previous study’s results, the ideal design for internal devices should consider building MRI-compatible internal/implantable devices. This is due to the high reliance on this imaging method for a myriad of secondary complications that people with SCI may experience across their lifespan [30].

Finally, this study has provided valuable insights into understanding bladder and bowel management in Australia and opens up several new pathways for potential future research to develop external and internal nerve stimulation devices to improve their bladder and bowel function in people with SCI. Further research would need to focus on consumer’s perspectives of specific devices as they become available, so that further modifications can meet consumer needs and priorities and be adopted successfully by people with SCI.

### Study limitations

This study’s limitations included the difficulty to evaluate the wider geographical representation of people with SCI across Australia, which may limit the comparison of results to the general SCI population. However, the study’s responses provided an indication of the willingness of people surveyed to accept and consider the use of nerve stimulation devices.This study’s limitations also include the small number of participants, a non-randomised selection of individuals with SCI to be included, and a lack of validated measures used.

## Conclusion

People with SCI living in Australia reported about their current bladder and bowel routine, needs, priorities as well as willingness to adopt nerve stimulation devices. Almost all of respondent’s stated that they would be willing to adopt both internal and external nerve stimulation devices if it meant improvement in overall independence in bladder and/or bowel management. Furthermore, implementing nerve stimulation devices may reduce medical complications related to bladder and/or bowel function. This survey has highlighted the importance of tailoring research specifically to identify the needs, priorities and goals of people with SCI in Australia. The findings highlighted the potential role in which nerve stimulation can have in addressing bladder and bowel dysfunction in people with SCI, and have also identified that there was a need for Australian physiotherapists evaluate their role in bladder and bowel dysfunction.

### Supplementary information

Copy of Qualtrics survey

Supplementary information for survey

## Data Availability

The datasets generated during and/or analysed during the current study are not publicly available due the data being part of a university research assignment only but are available from the corresponding authors on reasonable request.
