# Supplementary material for: Priorities, needs and willingness of use of nerve stimulation devices for bladder and bowel function in people with spinal cord injury (SCI): an Australian survey
Source: Spinal Cord Ser Cases. 2024 Mar 21;10:15. doi: 10.1038/s41394-024-00628-3 (PMC10957911; doi:10.1038/s41394-024-00628-3)
Supplement: Supplementary file 1 — Copy of Qualtrics survey [file 41394_2024_628_MOESM1_ESM.pdf]

RESEARCH WITH IMPACT

Information sheet and Consent

.

INFORMATION SHEET

BE WHAT YOU WANT TO BE
cqu.edu.au

Nerve stimulation for Neurogenic Bladder and Bowel Dysfunction in People with Spinal Cord Injury

Project Overview

This project is being completed by a group of physiotherapy students at CQUniversity, under the supervision of Dr Vanesa Bochkezanian. The project is about the issues that people with spinal cord injury (SCI) face with their bowel and bladder functions. We would like to know what your perceptions are around using nerve stimulation devices to help manage these problems.

Participation Procedure

You are invited to participate in an anonymous online survey which will take 20-30 minutes to complete. Participation will not affect your association with the University. Our aim is to find out if participants would be interested in using nerve stimulation technology to manage their bladder and bowel. Nerve stimulation technology provides precise, controllable (immediate on-off) nerve stimulation to the bladder and bowel muscles using small electrical signals to improve their function in both men and women.

Benefits and Risks

This survey may offer no direct benefit to participants. It offers information on available options and is expected to benefit people living with SCI, the wider community and the Physiotherapy field. We thank you for giving your time to participate. This survey should not

provoke any undue discomfort. However, if you are concerned, please consider viewing the support available at www.lifeline.org.au or contacting your General Practitioner.

Confidentiality / Anonymity

The survey will not collect any identifying information; your responses will be anonymous. Data will be securely stored for fifteen years after the completion of the research in accordance with the CQUniversity Code of Conduct for Research.

Outcome

The results of this research will be incorporated into an assessable piece by the students in the form of a dissertation or report. Additionally, a journal article or conference paper may arise out of this work.

Consent

Your consent to participate in this project will be obtained through your agreement to the Electronic Consent below.

Right to Withdraw

Your participation in this survey is voluntary. You may withdraw at any time prior to completing the survey by simply closing the browser. Information cannot be deleted after the survey is submitted as no identifiable information is collected and all responses are anonymous.

Feedback

This is part of an Honours project study with aim for future publication. A summary of the project will only be available after this study has been published in a peer reviewed journal.

Questions/ Further Information

If you have any questions about this project, please contact the Project Supervisor Dr Vanesa Bochkezanian via v.bochkezanian@cqu.edu.au. Please contact Central Queensland University's Research Division (Tel: 07 4923 2603; E-mail: ethics@cqu.edu.au) should there be any concerns about the nature and/or conduct of this research project. This project has been approved by the CQUniversity Human Research Ethics Committee, approval number 22316.

Devices

These devices are available in Australia and regulated by the Therapeutic Goods Act. However, these devices have not been validated and are not yet available to be used in people with spinal cord injuries (SCI) in Australia. This is first stage research to gather information about the potential benefits and risks of using nerve stimulation for bladder and bowel dysfunction in people with SCI living in Australia. This research study will inform the

next steps in this research area. Should you have any questions about this research study and any future research development in this area, you can contact the Chief Investigator: Dr Vanesa Bochkezanian (v.bochkezanian@cqu.edu.au).

<u>External Nerve Stimulation Devices</u>

The image shown below is an external nerve stimulation device, where electrodes are placed on the skin in different locations, which will achieve different effects in the body, with the ability to be taken on and off.

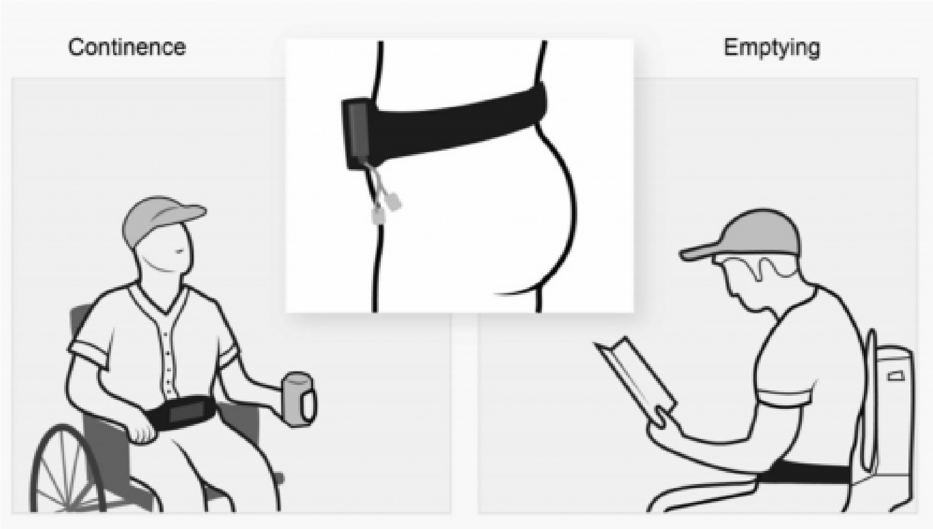

<u>Internal Nerve Stimulation Devices</u>

The image shown below is an Internal device that has electrodes surgically placed within the body. This also comes with a wireless hand-held controller that has the ability to turn the device on-off or to recharge the battery. This device can also have the electrodes placed in different areas to achieve different responses from the body.

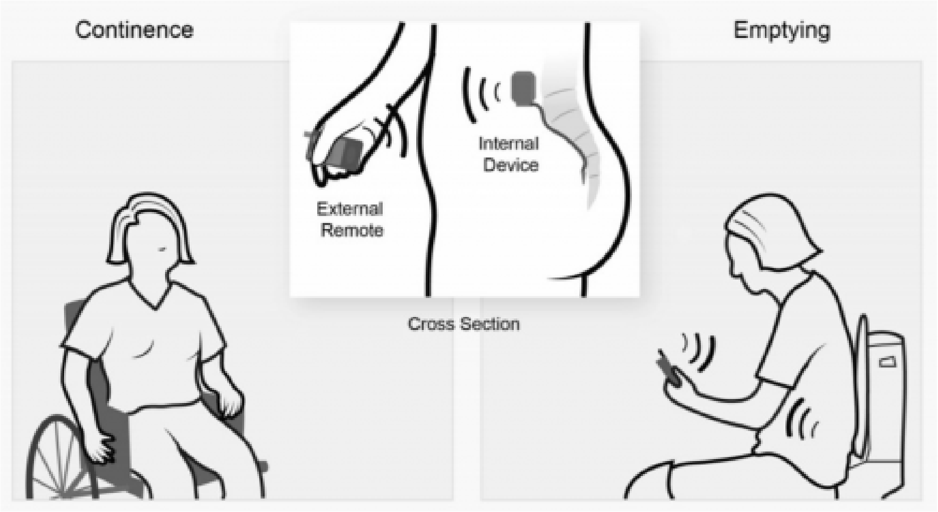

Potential Benefits of Nerve Stimulation Devices

Bladder function

- Emptying your bladder through your urethra without catheters
- Urinary continence (no leakage) without leg bags, absorbent pads, or other collection devices
- Manage your bladder without help from others
- Manage your bladder without daily medications
- Know when your bladder needs to be emptied
- Reduced medical complications such as urinary tract infections (UTIs), autonomic dysreflexia, bladder stones, or kidney disease.

Bowel function

- Reduced time required to empty your bowels
- Faecal continence (no leakage or bowel accidents)
- Greater predictability in bowel routine, including deciding where and when to empty your bowels
- Manage your bowels without help from others
- Know when your bowel needs to be emptied
- Reduced medical complications such as constipation, physical discomfort, or autonomic dysreflexia

Potential Risk and Inconveniences of all Nerve Stimulation Devices

- Unnatural although non-painful sensations from stimulation
- The battery will need to be recharged on a regular basis at home
- Adapting the device into your current care routine for bowel/ bladder
- Periodically returning to your doctor for follow up evaluations of the device

External Systems (outside of the body)

- Electrodes connected to the device will be in contact with your skin under your clothing
- On a daily basis you will have to take the device on-off

Internal Systems (surgically implanted in the body)

- Approx. 1 week of post-operative recovery
- 20% chance of temporary, post-surgical pain
- 2% chance of a post-surgical infection that requires treatment
- 50% chance of a problem with the implant that could be corrected without surgery (e.g. reprogramming)

- 20% chance of a problem with the implant that could require a revision surgery where you will stay in the hospital for a few days
- 4% chance of a problem with the implant that will lead to surgical removal of the whole system (e.g. wide-spread infection in your body)
- Battery replacement every 5 years, in a surgical procedure where you can go home the same day
- Not being able to have an MRI for life because of the implanted device

ELECTRONIC CONSENT:
Clicking on the "I Agree" button below indicates that you:

- Have a spinal cord injury (SCI) and bladder and/or bowel issues, are over 18 years old, and are an Australian permanent resident or citizen;
- Have read and understand the information above, and are agreeing to participate in the survey;
- Are aware that the data collected in the survey will be stored and analysed;
- Understand that your participation or non-participation in the research project will not affect your association with CQUniversity;
- Understand the research findings will be included in the researcher's publication(s) on the project and this may include conferences and articles written for journals and other methods of dissemination stated in the Information above;
- Understand that to preserve anonymity and maintain confidentiality of participants that fictitious names may be used in any publication(s).

○ I Agree
○ I Disagree

Q47.

INFORMATION SHEET

CQUniversity AUSTRALIA

BE WHAT YOU WANT TO BE
cqu.edu.au

Nerve stimulation for Neurogenic Bladder and Bowel Dysfunction in People with Spinal Cord Injury

Project Overview

This project is being completed by a group of physiotherapy students at CQUniversity, under the supervision of Dr Vanesa Bochkezanian. The project is about the issues that people with spinal cord injury (SCI) face with their bowel and bladder functions. We would like to know what your perceptions are around using nerve stimulation devices to help manage these problems.

Participation Procedure

You are invited to participate in an anonymous online survey which will take 20-30 minutes to complete. Participation will not affect your association with the University. Our aim is to find out if participants would be interested in using nerve stimulation technology to manage their bladder and bowel. Nerve stimulation technology provides precise, controllable (immediate on-off) nerve stimulation to the bladder and bowel muscles using small electrical signals to improve their function in both men and women.

Benefits and Risks

This survey may offer no direct benefit to participants. It offers information on available options and is expected to benefit people living with SCI, the wider community and the Physiotherapy field. We thank you for giving your time to participate. This survey should not provoke any undue discomfort. However, if you are concerned, please consider viewing the support available at www.lifeline.org.au or contacting your General Practitioner.

Confidentiality / Anonymity

The survey will not collect any identifying information; your responses will be anonymous. Data will be securely stored for fifteen years after the completion of the research in accordance with the CQUniversity Code of Conduct for Research.

Outcome

The results of this research will be incorporated into an assessable piece by the students in the form of a dissertation or report. Additionally, a journal article or conference paper may arise out of this work.

Consent

Your consent to participate in this project will be obtained through your agreement to the Electronic Consent below.

Right to Withdraw

Your participation in this survey is voluntary. You may withdraw at any time prior to completing the survey by simply closing the browser. Information cannot be deleted after the survey is submitted as no identifiable information is collected and all responses are anonymous.

Feedback
This is part of an Honours project study with aim for future publication. A summary of the project will only be available after this study has been published in a peer reviewed journal.

Questions/ Further Information
If you have any questions about this project, please contact the Project Supervisor Dr Vanesa Bochkezanian via v.bochkezanian@cqu.edu.au. Please contact Central Queensland University's Research Division (Tel: 07 4923 2603; E-mail: ethics@cqu.edu.au) should there be any concerns about the nature and/or conduct of this research project. This project has been approved by the CQUniversity Human Research Ethics Committee, approval number 22316.

Devices
These devices are available in Australia and regulated by the Therapeutic Goods Act. However, these devices have not been validated and are not yet available to be used in people with spinal cord injuries (SCI) in Australia. This is first stage research to gather information about the potential benefits and risks of using nerve stimulation for bladder and bowel dysfunction in people with SCI living in Australia. This research study will inform the next steps in this research area. Should you have any questions about this research study and any future research development in this area, you can contact the Chief Investigator: Dr Vanesa Bochkezanian (v.bochkezanian@cqu.edu.au).

External Nerve Stimulation Devices
The image shown below is an external nerve stimulation device, where electrodes are placed on the skin in different locations, which will achieve different effects in the body, with the ability to be taken on and off.

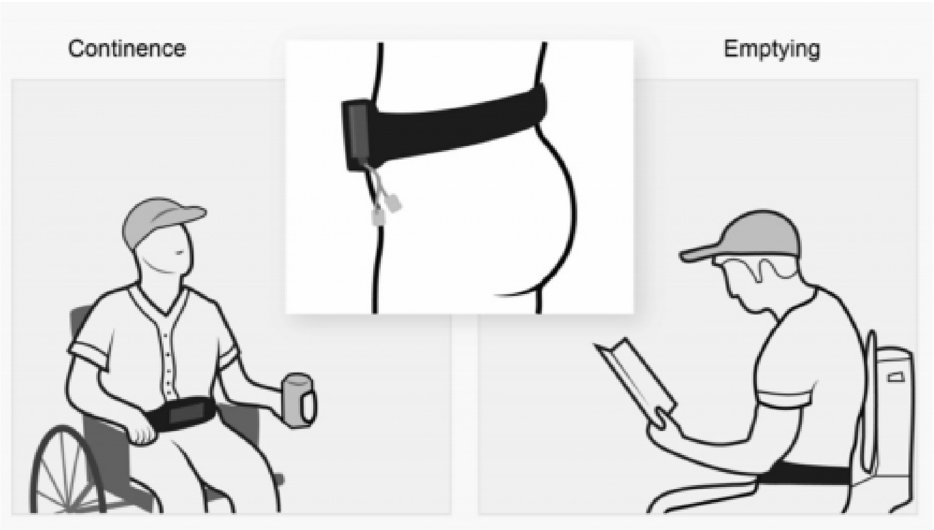

Internal Nerve Stimulation Devices

The image shown below is an Internal device that has electrodes surgically placed within the body. This also comes with a wireless hand-held controller that has the ability to turn the device on-off or to recharge the battery. This device can also have the electrodes placed in different areas to achieve different responses from the body.

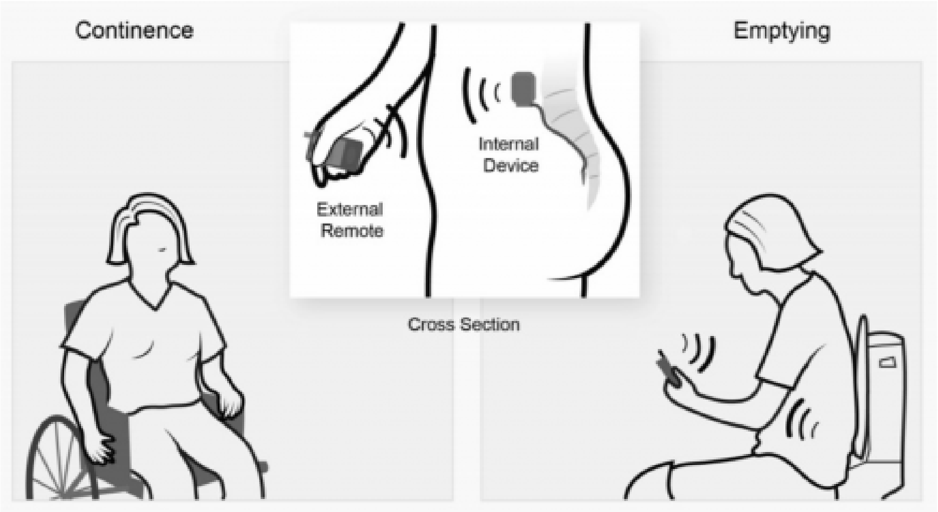

Potential Benefits of Nerve Stimulation Devices

Bladder function

- Emptying your bladder through your urethra without catheters
- Urinary continence (no leakage) without leg bags, absorbent pads, or other collection devices
- Manage your bladder without help from others
- Manage your bladder without daily medications
- Know when your bladder needs to be emptied
- Reduced medical complications such as urinary tract infections (UTIs), autonomic dysreflexia, bladder stones, or kidney disease.

Bowel function

- Reduced time required to empty your bowels
- Faecal continence (no leakage or bowel accidents)
- Greater predictability in bowel routine, including deciding where and when to empty your bowels
- Manage your bowels without help from others
- Know when your bowel needs to be emptied
- Reduced medical complications such as constipation, physical discomfort, or autonomic dysreflexia

Potential Risk and Inconveniences of all Nerve Stimulation Devices

- Unnatural although non-painful sensations from stimulation
- The battery will need to be recharged on a regular basis at home
- Adapting the device into your current care routine for bowel/ bladder
- Periodically returning to your doctor for follow up evaluations of the device

External Systems (outside of the body)

- Electrodes connected to the device will be in contact with your skin under your clothing
- On a daily basis you will have to take the device on-off

Internal Systems (surgically implanted in the body)

- Approx. 1 week of post-operative recovery
- 20% chance of temporary, post-surgical pain
- 2% chance of a post-surgical infection that requires treatment
- 50% chance of a problem with the implant that could be corrected without surgery (e.g. reprogramming)
- 20% chance of a problem with the implant that could require a revision surgery where you will stay in the hospital for a few days
- 4% chance of a problem with the implant that will lead to surgical removal of the whole system (e.g. wide-spread infection in your body)
- Battery replacement every 5 years, in a surgical procedure where you can go home the same day
- Not being able to have an MRI for life because of the implanted device

<u>ELECTRONIC CONSENT:</u>
Clicking on the "I Agree" button below indicates that you:

- Have a spinal cord injury (SCI) and bladder and/or bowel issues, are over 18 years old, and are an Australian permanent resident or citizen;
- Have read and understand the information above, and are agreeing to participate in the survey;
- Are aware that the data collected in the survey will be stored and analysed;
- Understand that your participation or non-participation in the research project will not affect your association with CQUniversity;

- Understand the research findings will be included in the researcher's publication(s) on the project and this may include conferences and articles written for journals and other methods of dissemination stated in the Information above;
- Understand that to preserve anonymity and maintain confidentiality of participants that fictitious names may be used in any publication(s).

○ I Agree
○ I Disagree

Demographic Information

. In this section there will be questions asked about your demographic information. Please select the most appropriate answer. If you make a mistake that is okay, simply un-select your current response and select the new answer. There are no right or wrong answers.

Dem 1. Do you understand what is written in the English language on the information sheet and agree to participate?

○ Yes
○ No

Dem 2. Has someone assisted you to complete this survey?

○ Yes
○ No

Dem 3. What is your gender?

○ Male
○ Female
○ Other
○ Prefer not to specify

Dem 4. What is your age?

○ 18-30
○ 31-45
○ 46-60
○ 61-75
○ 76+

Dem 5. What is your geographical location in Australia?

- ◯ Metropolitan (e.g Sydney, Brisbane) - Population 100,000 or more
- ◯ Non- Metropolitan (e.g Rockhampton, Mackay) - population less than 100,000

Dem 6.

Has your bowel been impacted by a traumatic or non- traumatic spinal cord injury?

[▾]

Dem 7. Has your bladder been impacted by a traumatic or non- traumatic spinal cord injury?

[▾]

Dem 8. How long have you had your spinal cord injury?

- ◯ Less than 1 year
- ◯ 1 - 5 years
- ◯ 6 - 10 years
- ◯ 11 - 15 years
- ◯ 16 - 20 years
- ◯ Over 20 years

Dem 9. What level was your spinal cord injury?

- ☐ Cervical - C1-4
- ☐ Cervical - C5-8
- ☐ Thoracic OR Lumbar OR Sacral - T1-S5

Dem 10. How severe is your impairment?

- ☐ Complete lack of motor and sensory function below the level of injury, including the anal area
- ☐ Some sensation below the level of the injury, including anal sensation
- ☐ Less than 50% of the muscles that are working below the level of injury, are strong enough to move against gravity
- ☐ More than 50% of the muscles that are working below the level of injury, are strong enough to move against gravity
- ☐ Unsure

Dem 11. Which statement below applies to you for transfers?

- ○ I require no assistance with transfers (eg: moving from bed to chair)
- ○ I require minimal assistance with a device (eg: slide board) or caregiver to complete transfers
- ○ I require full assistance with all transfers

Dem 12. Which statement applies to your hand function?

- ○ Complete ability to perform hand movements and tasks
- ○ Ability to perform some hand movements and tasks
- ○ No ability to perform hand movements or tasks

Dem 13. How often do you normally leave the house for employment, education or social activities?

- ○ Daily
- ○ Weekly
- ○ Fortnighty
- ○ Monthly
- ○ Never

Dem 14. Do you currently have any problems with transport to access healthcare services?

- ○ No
- ○ [ ] Yes (please specify)

Dem 15. Does your financial status interrupt accessing routine health care and medical supplies?

- ○ Yes
- ○ No
- ○ Would rather not say

Dem 16. Where do you source the information to assist with your bowel and/or bladder management plan?
(select all that apply)

- ☐ Health care providers
- ☐ Educational organisation
- ☐ Support groups
- ☐ Internet

- [ ] Friends
- [ ] Information sessions
- [ ] ____________ Other

Dem 17. Which health professionals help you manage your bowel and/ or bladder? (select all that apply)

- [ ] Physiatrist
- [ ] Neurologist
- [ ] Neurosurgeon
- [ ] Urologist
- [ ] Gastroenterologist
- [ ] Primary Care Physician (GP)
- [ ] Nurse
- [ ] No access to a health professional
- [ ] ____________ Other (please specify)

Bladder Management

.

This section asks about your bladder management. Some questions may ask you to select all answers relevant to you or rank the answers in order of importance.

Bladder 1. How do you currently manage your bladder? (select all that apply)

- [ ] I do not use any equipment
- [ ] Indwelling (Foley) catheter via urethra
- [ ] Condom catheter
- [ ] Suprapubic catheter
- [ ] Electrical stimulation
- [ ] Intermittent catheterisation via urethra
- [ ] Intermittent catheterisation via abdomen
- [ ] Bag on abdomen
- [ ] Absorbent pads or diapers
- [ ] ____________ Other

Bladder 2.

On a daily basis how many times would you empty your bladder?

Bladder 3. Do you take daily medication to manage your bladder?

Yes No I don't know

Bladder 4. When emptying your bladder how much assistance do you require?

No assistance

Some assistance

Full assistance

Bladder 5. If you experience an episode of urine incontinence (bladder accident), how much assistance do you require?

- I do not experience urinary incontinence
- No assistance
- Some assistance
- Full assistance

Bladder 6. Does your body give a conscious indication of when your bladder needs to be emptied?

- Yes
- No

Bladder 7. If you are aware when you need to empty your bladder, is there enough time to avoid an episode of urine incontinence?

- Not Applicable - I do not feel when my bladder is full
- Yes
- No

Bladder 8. In the past 12 months as a result of your current bladder management have you experienced any of the following complications? (select all that apply)

- ☐ I do not experience complications from my bladder management
- ☐ Clogged catheter
- ☐ Urinary tract infection
- ☐ Urinary incontinence
- ☐ Bladder or kidney stones
- ☐ Kidney disease, kidney failure or both
- ☐ Autonomic Dysreflexia (AD)
- ☐ ________ Other (please specify)

Bladder 9. Please select the most applicable answer to the statements below

Are daily routines disrupted because of your bladder? [ ]

Is the ability to participate in education and/ or employment interrupted because of your bladder? [ ]

Does your bladder interfere with activities with family, neighbours or social groups? [ ]

Bladder 10. In regards to the future, list the following benefits in order of importance from 1 to 6

[ ] Emptying your bladder through your urethra without catheters, assuming that transfers are not a problem

[ ] Managing or improving urinary continence

[ ] Independently managing your bladder

[ ] Bladder management without medications

[ ] Sensing when your bladder needs to be emptied

[ ] Improvement of medical complications associated with your bladder and kidneys

Bowel Management

.

This section asks about your bowel management. Some questions may ask you to select all answers relevant to you or rank the answers in order of importance.

Bowel 1. What is the average duration for you to complete your bowel routine?

Bowel 2. How do you currently manage your bowel? (select all that apply)

- ☐ Digital stimulation
- ☐ Manual evacuation
- ☐ Laxatives
- ☐ Special diet
- ☐ Implanted devices
- ☐ External adaptive devices
- ☐ Suppositories
- ☐ Enemas
- ☐ Colostomy bag
- ☐ Other

Bowel 3. Do you use medication daily to manage your bowel?

Yes ○ No ○ Not sure ○

Bowel 4. When you are emptying your bowel do you require any assistance?

No assistance ○

Some assistance ○

Full assistance ○

Bowel 5. If an episode of faecal incontinence (bowel accident) occurs, how much assistance do you require ?

- ○ I do not experience faecal incontinence
- ○ None
- ○ A moderate amount
- ○ A lot

Bowel 6. Does your body give a conscious indication of when a bowel movement is required?

Yes ○ No ○

Not applicable - I use a colostomy bag ○

Bowel 7. If you sense when your bowel needs to be emptied, do you usually have enough time to avoid incontinence?

- ○ N/A - I do not feel when my bowel is full
- ○ Yes
- ○ No

Bowel 8. In the past 12 months as a result of your current bowel management have you experienced any of the following complications? (select all that apply)

- ☐ I do not experience complications with my bowel
- ☐ Faecal incontinence
- ☐ Constipation
- ☐ Autonomic dysreflexia (AD)
- ☐ Hemorrhoids
- ☐ Bleeding
- ☐ Loose stool
- ☐ Complications with colostomy stoma
- ☐ ________ Other

Bowel 9. Please select the most applicable answer to the statements below

Are daily routines disrupted because of your bowel?	Is the ability to participate in education and/ or employment interrupted because of your bowel?	Does your bowel interfere with activities with family, neighbours or social groups?

Bowel 10. In regards to the future, list the following benefits in order of importance from 1 to 7

☐ Reducing time required for your bowel routine and reducing constipation

☐ Your bowel routine having predictability

☐ Maintaining or improving faecal continence (no leakage or bowel accidents)

☐ Independence in bowel management

☐ Reduction of medical complications involving the bowel

☐ Managing your bowel without daily medications

☐ Sense when your bowel needs to be emptied

Options for Nerve Stimulation

.

The following questions will be asked around nerve stimulation systems and what your opinion is on the potential risks and benefits.

NS 1. What would be your main concerns around using an external nerve stimulation device? Please rank the following in order from 1 - 6 with 1 being the most concerning to you.

☐ Wearing a device with wires connecting to electrodes on the skin under the clothes

☐ Having to put the device on and take the device off as needed on a daily basis

☐ Unnatural, but not painful, sensations from stimulation

☐ Battery recharging at home on a regular basis

☐ Learning how to use the device with your current bladder or bowel management strategies

☐ Returning to clinic periodically for follow up device evaluation

NS 2. What would be your main concerns around using an internal (implanted) nerve stimulation device? Please rank the following in order from 1 - 12 with 1 being the most concerning to you.

☐ Approximately 1 week of post-operative recovery

☐ 20% chance of experiencing temporary, post-surgical pain

☐ 2% chance of experiencing a post-surgical infection that requires treatment

☐ 50% chance of experiencing a problem with the implant that could be corrected without requiring surgery (e.g. reprogramming)

☐ 20% chance of experiencing a problem with the implant that could require a revision surgery where you will stay in the hospital for a few days

☐ 4% chance of experiencing a problem with the implant that will lead to surgical removal of the whole system (e.g. a wide-spread infection in your body)

☐ Having the battery replaced every 5 years, in a surgical procedure where you can go home the same day

☐ Not being able to have an MRI for life because of the implanted device

☐ Unnatural, but not painful, sensations from stimulation

☐ Battery recharging at home on a regular basis

☐ Learning how to use the device with your current bladder or bowel management strategies

☐ Returning to clinic periodically for follow-up device evaluations

NS 3.

How likely would you be to use the external nerve stimulation device to achieve the outcomes below?

	External Device					
	Not Likely		Somewhat Likely		Very Likely	N/A
Pass urine normally without using catheters	○	○	○	○	○	○
Improve urinary continence without using collection devices	○	○	○	○	○	○
Predictability and reduced duration of bowel routine	○	○	○	○	○	○
Improvement of faecal continence with a decrease in leakage and incontinence	○	○	○	○	○	○
Improvement of overall independence for bladder/ bowel management	○	○	○	○	○	○
Cease daily medication for bowel and/or bladder management	○	○	○	○	○	○
Reduce medical complications that are related to bowel/ bladder function	○	○	○	○	○	○

NS 4.

Knowing the risks and benefits would you be more accepting of the internal (implanted) device if it achieved the outcomes below?

	Internal (implanted) device					
	Not likely		Somewhat likely		Very Likely	N/A
Pass urine normally without the use of catheters	○	○	○	○	○	○
Improve your urinary continence without using collection devices	○	○	○	○	○	○
Predictability and duration of your bowel routine	○	○	○	○	○	○
Improvement of fecal continence with a decrease in leakage and incontinence	○	○	○	○	○	○
Improvement of overall independence for bladder/ bowel management	○	○	○	○	○	○
Cease daily medication for bowel and/or bladder management	○	○	○	○	○	○
Reduce medical complications that are related to bowel/ bladder function	○	○	○	○	○	○

Powered by Qualtrics
