# Supplementary material for: Priorities, needs and willingness of use of nerve stimulation devices for bladder and bowel function in people with spinal cord injury (SCI): an Australian survey
Source: Spinal Cord Ser Cases. 2024 Mar 21;10:15. doi: 10.1038/s41394-024-00628-3 (PMC10957911; doi:10.1038/s41394-024-00628-3)
Supplement: Supplementary file 2 — Supplementary information for survey [file 41394_2024_628_MOESM2_ESM.docx]

Questions within the survey were arranged into four categories: demographics, bladder function, bowel function and attitudes toward nerve stimulation. Questions were divided into two separate sections for bladder and bowel function and management; however the questions were similar for both sections. Some of these questions were related to the current bladder and bowel management and its challenges; level of interference bladder and bowel management may have in activities of daily living, social and professional activities. Additionally, these questions aimed to determine how respondents ranked bladder and bowel functions that they would like to have improved or restored, and these forced rank questions also identified respondents’ priorities for improving or restoring bladder and bowel function.

One of the main objectives of this study was to identify respondents’ willingness to accept external or internal nerve stimulation devices to improve or restore bladder and bowel functions. For this purpose, embedded in the survey, an information sheet was included, in which a generalised definition of a nerve stimulation device including potential external and implanted versions was presented. This information included known risks with estimated rates of occurrence based on the literature for similar technologies [1], which allowed respondents to express and rate levels of concern for each of these risks. This information aimed to help respondents determine their willingness to accept these devices to improve or restore bladder and bowel functions.

Reference:

1. Bourbeau D, Bolon A, Creasey G, Dai W, Fertig B, French J et al. Needs, priorities, and attitudes of individuals with spinal cord injury toward nerve stimulation devices for bladder and bowel function: a survey. Spinal Cord. 2020; 58(11):1216-1226.  https://doi.org/10.1038/s41393-020-00545-w
